# The Western Lancet

**Published:** 1842-08

**Authors:** 


THE WESTERN LANCET.

The editor of the Western Lancet, published at Cincinnati, evinces much surprise at an editorial article, entitled "Western Periodicals," in our June number, and seizes the first moment of returning tranquillity to read what he considers, no doubt, a short lecture, to the press generally and to our senior editor and this Journal in particular. We regret not having space for the whole of his remarks, as we would like the reader to see what a raw-head-and-bloody-bones a man's distempered fancy can create out of nothing.

In speaking of the periodicals published in the West, we made the following observations: "These journals are in the interests of the three medical schools in the West; and may be expected, respectively, to advance those interests by every means consistent with *truth* and a *generous* rivalship." The imputation here conveyed, or as the Lancet is disposed to term it in *elegant* phrase, "the specialty in medical partisanship here assigned," seems actually to have frightened our contemporary. He evinces as much sensibility in regard to it, as if it were a matter of life and death, though, what there is in the imputation to excite so much feeling, is beyond our ken. Is it so heinous a matter for a journal to be devoted to the interests of a school of medicine, and to advance those interests by all *honorable* means? Is it inconsistent with a proper independence? does it presuppose, or involve in any way, a sacrifice of principle? The sort of holy horror with which he views the bare idea of any connection with the Medical College of our sister State, is calculated to excite suspicion; it might easily lead a stranger to suppose there was something wrong about that institution, either that its interests were totally opposed to those *general* ones which, in his immense liberality, the editor of the Lancet has taken under his especial guardianship, or that there was some upas-like influence, some pestilential atmosphere about the building that was absolutely fatal to every thing so unfortunate as to be in its neighborhood. Yet we cannot but reflect that once upon a time we dwelt within its shadow, almost under its very eaves; that each morning its red front glared on our half-opened eyes, and each evening its burnished windows greeted our backward gaze; and, forsooth, the grass waved as luxuriantly, playing with the iron railing that encloses it, and the trees that adorn its front tossed their vigorous boughs and danced as joyously in the passing breeze, as in the green fields and forests beyond. Not being disposed, however, to assign the Lancet a position that it does not wish to occupy, we correct the error into which we inadvertently fell, and give the editor's disclaimer in his own language, viz. "The Medical College of Ohio does not require our aid, and if it did, we do not feel competent to render such service." As we cannot question the truth of the former clause, so we certainly shall not doubt that of the latter.

The editor's fancy is not only distempered, raising up "gorgons and chimeras dire," but his mental vision is likewise morbidly acute. He is not only able to see as far into a mill-stone as the man that picks it, but he can actually see further; for he has found in the editorial referred to, a censure on all attempts to establish new medical journals! This singular *presbyopia* might be explained by supposing some votary of the magic-like art of mesmerism had been practising on his delicate susceptibility, if it did not evince a degree of perspicacity, a mental clear-sightedness to which the phenomena of *clair-voyance* afford no sort of parallel. He has succeeded so well in exposing what he denominates our "partial and erroneous view of the subject," that we dislike to disturb his self-complacency by showing that such "view" never had any existence save in his own diseased imagination. We hope, however, that upon "sober second thought," he will discover we have somewhat more of liberality and of magnanimity than he now gives us credit for.

The Lancet has a good deal to say about *parties* and *party feuds*, *party interests*, *partisan feelings*, *&c. &c.*, all which *may* be very fine, but, "as we understand it," is about the merest twattle imaginable, much better suited to the columns of some obscure political sheet, than to the pages of a medical journal professing and aiming to be respectable. It is a common observation, too, that those who are *loudest* in their condemnation of such things, who apparently hold them in the *most* abhorrence, are ever the first to submit to their sway.

We do not mean to say at all that such is the case in the present instance; we would fain believe it is not, and are willing to think our contemporary honest in his professions; but we beg him to bear the fact in mind, in his future lucubrations, to the end that no such impression of him be entertained. C.

